# Evaluation of Stages, Treatment Protocols, and Outcomes of Colorectal Cancer among West Bank Patients

**DOI:** 10.3390/jcm13082284

**Published:** 2024-04-15

**Authors:** Ibrahim O. Sawaid, Abraham O. Samson, Rowa Al-Ramahi

**Affiliations:** 1 Azrieli Faculty of Medicine, Bar Ilan University, Safed 1311502, Israel; avraham.samson@biu.ac.il; 2 Faculty of Graduate Studies, An-Najah National University, Nablus P400, Palestinian Territory; rowa@naja.ac.pl

**Keywords:** colorectal cancer, colon cancer, rectal cancer, healthcare, West Bank

## Abstract

**Background:** Colorectal cancer (CRC) is the second most widespread cancer among Palestinian patients. As cancer care improves in hospitals across the West Bank, services like palliative care, targeted therapy, bone marrow transplantation, and individualized therapy are still limited. This study aimed to assess the CRC stages, treatment protocols, and survival rates of patients in the West Bank. **Methodology:** This retrospective study collected data from the medical records of Al-Najah University Hospital (NUH), which specializes in the care of cancer patients. Patients with confirmed CRC (stages I–IV) undergoing surgical or medical treatment were included in the study. Data collection was standardized by using a data collection form to gather information from the medical records included in the study. All statistical analyses were performed using SPSS (version v27), and survival was assessed using a regression analysis of the number of days from the time of diagnosis to the most recent visit against the type of treatment (e.g., surgery, chemotherapy, radiotherapy). **Results:** A sample of 252 patients with CRC from NUH was collected, including 143 males and 109 females aged between 27 and 86 years, with the average age being 60.6 ± 11.4 years. The sample included 183 patients (72.6%) diagnosed with colon cancer only, 29 patients (11.5%) diagnosed with rectal cancer only, and 40 patients (15.9%) diagnosed with both. Diagnosis took place at CRC stage I for 3 patients (1.2%), stage II for 33 patients (13.1%), stage III for 57 patients (22.6%), and stage IV for 159 patients (63.1%). Surgery was the most prevailing mode of treatment for 230 patients (91.3%), while 227 patients (90.1%) received chemotherapy treatment, and 38 patients (15.1%) received radiotherapy. Of the 252 patients, 40 patients (15.8%) received FOLFOX (i.e., folinic acid, fluorouracil, oxaliplatin), and 25 patients (9.9%) received FOLFIRI (i.e., folinic acid, fluorouracil, irinotecan), while the 187 remaining patients (74.2%) were treated with capecitabine, oxaliplatin, bevacizumab, cetuximab, regorafenib, cisplatin, etoposide, gemcitabine, or a combination thereof. The sample was categorized into six outcomes: (1) death, (2) cure, (3) disease progression, (4) disease recurrence, (5) under-treatment, and (6) unknown. Mortality was high, with 104 patients (41.3%) dying within a short time after diagnosis, and may have been attributable to delayed diagnosis. Surgical treatment had a positive impact on increasing the survival years, and it was significant (*p* = 0.033). **Conclusions:** A high percentage of patients were diagnosed in advanced CRC stages. The treatment modes were adopted from general international guidelines; however, the cure rates were low, and mortality was high. More studies need to be undertaken to investigate the actual application of chemotherapy protocols, and survival would benefit from the involvement of clinical pharmacists in the chemotherapy protocol selection, dosing, frequency, and follow-up. The present study advocates for greater public awareness of CRC and attests to the merits of screening by primary care professionals, which can help to avoid this serious illness and to promote a better prognosis.

## 1. Introduction

Colorectal cancer (CRC) is one of the most prevalent forms of cancer [1]. Over the past few decades, CRC incidence has increased significantly, as CRC is now no longer the third most prevalent cancer behind breast cancer and lung cancer [2]. In 2000, less than a million (i.e., 945,000) new cases were diagnosed, accounting for 9.4% of all cancers and 492,000 cancer deaths [3]. In 2007, CRC became the second most prevalent cause of cancer mortality among men and women globally, with an annual incidence of roughly 1 million cases and more than 500,000 deaths [4]. In 2008, more than 1 million new cases were diagnosed, and CRC remains the second most prevalent cause of cancer mortality, behind lung cancer [5]. CRC is most common in high-income and industrialized areas like North America and Western Europe, as well as some Asian countries, including Japan and Singapore [6]. Notably, CRC is relatively uncommon in some Africa and Asian nations, with males being more affected than females [7]. During the last few decades, however, the prevalence of CRC has risen dramatically all across Asia. Furthermore, data from this region indicate that this prevalence has already approached that of the West, particularly among the more wealthy inhabitants [8].

The underlying causes of colorectal cancer are not completely understood. It is thought that several risk factors are involved in the development of CRC over a long period. The risk for colorectal cancer varies from country to country and even within countries; it varies among individuals based on several factors, such as diet, lifestyle, and hereditary factors. One study found that one or two generations of immigrants relocating from low-incidence CRC nations to high-incidence ones had a greater colorectal cancer burden [9]. Diet and lifestyle variables such as a low-fiber and high-fat diet, along with red meat and alcohol consumption, sedentary work, and cigarette smoking, are key modifiable etiologic causes. In addition, advanced age (50+), a family history of CRC, a personal history of CRC, adenoma, a genetic history of non-polyposis CRC syndrome, or inflammatory bowel disease of the colon, such as ulcerative colitis and Crohn’s disease, are all risk factors for CRC [9]. Notably, only ~5% of all CRC occurrences are caused by hereditary disorders. CRC develops in phases, with each step resulting in the transformation of a normal epithelial cell into an adenocarcinoma. Colonocytes with genetic mutations are first able to multiply at an abnormally high pace. As the process progresses, the cells develop malignant traits such as invasiveness and the ability to spread [10].

The American Cancer Society has recommended that everyone over the age of 45 be screened for CRC since most sporadic cases in industrialized nations occur in adults over this age. CRC screening allows for the cost-effective early diagnosis and treatment of an early-stage cancer. Individuals who have had frequent check-ups with fecal occult blood testing have been demonstrated to have a lower risk of CRC death [11,12]. Further reductions can be achieved by performing sigmoidoscopy and colonoscopy, followed by colonoscopic polypectomy. Many studies have shown that the obstacle to implementing a CRC screening program is limited by the knowledge of CRC, the embarrassing nature of the test, and the lack of physician recommendations [13].

In the West Bank, breast cancer, lung cancer, colon cancer, leukemia, and brain cancer account for 58.6% of cancer cases resulting in death among Palestinians, accounting for more than half of all cancer fatalities, according to statistics from the National Cancer Registry in the West Bank, a division of the Health Information Center [14]. In the West Bank, colon cancer is ranked number one among cancers affecting men, with a rate of 11.2%. However, among cancers affecting both genders, colon cancer is ranked as the second most common cancer type with a rate of 9.4%, followed by lung cancer, which is third with a rate of 8.7% [15]. The Palestinian Authority Ministry of Health official statistics revealed that the total number of cancer cases reported in the governorates of the West Bank was 3174 cases in 2019, an increase of 2.2% over the 2102 cases reported in 2018. In 2019, the incidence of cancer in the West Bank was 117.8 per 100,000 inhabitants. The number of new cases registered in 2019 was similar between the genders, with 1664 female cases (52.4%) and 1510 male cases (47.6%). In 2019, approximately 1095 cancer cases (34.5%) were registered in the >64 years age group, although this age group represented only 3.3% of the total population. While 1936 cases (61.0%) of the cases were recorded in the 15–64 age group, there were 143 cases (4.5%) in the <15 years old age group, although the percentage of this group was 38.4% of the total population [16].

In the West Bank, patients diagnosed with cancer are treated in the hospitals of the Palestinian Authority Ministry of Health, particularly at Al-Hussein Hospital in Beit Jala and Alwatani Hospital in Nablus, which are considered the main centers for cancer treatment in the West Bank. While epidemiologic studies are absent in the West Bank, it is mandatory to follow up on the treatment protocols, the risk factors behind disease development among the Palestinian population, and survival rates. To the best of our knowledge, there has been no study investigating the survival of colorectal cancer patients in the West Bank, and there is a shortage of studies exploring adherence to international therapeutic guidelines. Thus, the current study aimed to investigate colorectal cancer among the Palestinian population in the West Bank in terms of disease prevalence, survival rates, treatment protocols, and risk factors. Such studies will provide insights for healthcare providers into the Palestinian population, helping them to deliver optimal healthcare. The Palestinian Authority on healthcare has become increasingly important since the 1994 Oslo agreement, after the Palestinian Authority resumed control of healthcare organizations in the West Bank and Gaza Strip. The WHO and foreign donors support this administration, especially the US Government. Even though there have been major economic and social challenges in this area, the healthcare sector in the West Bank is one of the best among all Arab countries in terms of life expectancy and low maternal, infant, and child mortality rates [17].

In the West Bank, there are a limited number of hospitals that provide care for cancer patients. Radiation therapy and personalized oncology are only available at Augusta Victoria Hospital (Jerusalem), while bone marrow transplantation is only available at An-Najah University Hospital (Nablus). These hospitals also refer patients to other hospitals in Israel, Jordan, and Egypt. Some diagnostic tests are unavailable in the West Bank, such as PET-CT, and cases requiring these services are also referred to other hospitals [18]. Cancer care is improving in West Bank hospitals with time; however, services like palliative care, targeted therapy, bone marrow transplantation, and individualized therapy are still limited. This is due to many causes, including the lack of specialized physicians, shortages of drugs, etc. As the population grows, the cancer burden in the West Bank is likely to rise, adding more strain on the present healthcare system’s financial and technical resources [15].

To evaluate the accuracy of mortality data, and cancer mortality patterns in the West Bank, a recent study analyzed death certificates issued there. Notably, the highest rate of cancer mortality was associated with lung cancer among males (22.8%) and breast cancer among females (21.5%), followed by prostate cancer for males (9.5%) and by colon cancer for females (11.4%). The study concluded that the Palestinian mortality registry has improved over time [19].

This study aims to assess the colorectal cancer (CRC) treatment protocols and the survival of patients in the West Bank. Specifically, this study determines the distribution of cases according to gender, age, and the stages of colorectal cancer among West Bank patients. In addition, this study presents the common modes of treatment protocols used by physicians to treat patients with colorectal cancer in the West Bank. Finally, this study explores the relevant factors affecting CRC prognosis. As such, this study documents CRC survival and associated prognostic factors in the West Bank. It provides data on the treatments of choice, the treatment protocols, and the stages of disease among patients in the West Bank. Finally, the study analyzes treatment outcomes, which will help in evaluating the situation and developing policies in this field.

## 2. Methods

Study Design. This was a retrospective study carried out through data collection from medical records in a hospital that specializes in cancer patient care. The medical records of colorectal cancer (CRC) patients from An-Najah National University Hospital, Nablus (NUH), were reviewed during January and February of 2021. The time covered in the records was from January 2014 to February 2021. This hospital is a tertiary hospital for cancer treatment in the northern region of the West Bank. Only records of patients with confirmed colorectal cancer (stages I–IV) undergoing surgical or medical treatment were recorded.

Inclusion and exclusion criteria. The study included the following: (1) patients above 18 years, (2) males and females, (3) those with a confirmed diagnosis of CRC, and (4) patients with CRC who had received surgical treatment (resection, laparotomy, colectomy), radiotherapy, and/or chemotherapy. The exclusion criteria were as follows: (1) patients less than 18 years old, (2) pregnant patients, and (3) patients who did not receive any type of treatment.

According to annual statistical reports published by the PA Ministry of Health, the number of reported colorectal cancer cases every year in the West Bank is between 300 and 400; this study included the files of all patients who were treated at NUH who met the inclusion criteria.

Data collection. Considering the importance of data standardization for the internal validity of a study, data collection was standardized using a data collection form to gather information from included patients’ medical records (Supplementary Table S1). All clinical cases and their follow-up data were recorded. These data included gender, age at diagnosis, clinical symptoms, severe complications, location of the primary tumor, histological type, tumor differentiation, lympho-vascular invasion, depth of invasion, number of retrieved lymph nodes and metastatic lymph nodes, date of surgery, date of recurrence (if applicable), cause of recurrence (if applicable), date of death (if applicable), cause of death (if applicable), postoperative treatment, and date of follow-up.

Ethical considerations. The study protocol was authorized by NUH’s institutional review boards (IRB) (Permit No. Mas Sep/2020/3) before the initiation of the study. All information obtained from medical records was kept confidential, and only summarized data were presented in reports or publications. The maintenance of high-level objectivity in discussions and analyses carried out throughout the research was ensured.

Statistical analyses. Statistical analyses were performed using SPSS (version 21). Means and standard deviations were computed for continuous data. Frequencies and percentages were calculated for categorical variables. Categorical variables were compared using the χ-square test and Fisher test as appropriate. A *p*-value of less than 0.05 was considered to be statistically significant for all analyses.

## 3. Results

This section presents the results obtained from a sample of 252 patients with colorectal cancer (CRC) from Najah University Hospital (NUH). The medical records were obtained with approval from the Archive Department of NUH using the Medical Record System at NUH.

Patient demographics. The demographic data of the patients are shown in Table 1. Male patients were more prevalent than females, with a ratio of 1.31:1. The patients ranged in age between 27 and 86 years, with a mean age of 60.64 (±11.4) years; most of them were married, non-drinkers, and non-smokers. Notably, 46 (18%) of the patients were aged under 50 years old, attesting to the global concern regarding the increasing incidence of early-onset colorectal cancer [20]. Socio-demographic information, including data about age, gender, marital status, smoking, weight, height, blood group, education, nutrition, and work, was evaluated. Strikingly, the medical records suffered from scarce data about weight, height, blood group, education, nutrition, and work; hence, no meaningful results could be obtained from them.

About half of the 252 patients did not present with a history of any disease other than CRC. Data regarding the few comorbid diseases that were recorded are shown in Table 2. In some instances, more than one comorbid disease was recorded, giving rise to a range of medical histories. Notably, the reported comorbidities lack important history about inflammatory bowel diseases, allergies, etc.

Colorectal cancer (CRC) characteristics. Out of the 252 patients, 183 of the patients had colon cancer only (72.6%), 29 had rectal cancer only (11.5%), and the remaining 40 patients had both rectal and colon cancer (15.9%), or the tumor was located on the verge of colon and rectum. The stages of colorectal cancer, categorized using the I–IV and TNM systems, are shown in Table 3 and Table 4, respectively. A high percentage of patients (63.1%) were unfortunately in stage IV. Likewise, a high number of patients were diagnosed with advanced primary tumors with metastases and lymph node involvement.

In our χ-square analysis, there was no significant association between gender and stage (*p* = 0.553). In terms of rectal or colon cancer, there was also no significant association between gender and type of cancer (colon/rectal), as reflected by their *p*-values (0.539/0.965), as shown in Table 5.

Treatment strategies. The data collection form had specific questions regarding the CRC treatment. First, it asked about the strategy in general (surgery, radiotherapy, or chemotherapy), as shown in Table 6. Then, it asked about the chemotherapy protocol used to treat the patients in terms of the protocol used and the number of cycles of each protocol. Notably, most patients received a combination therapy including surgery and chemotherapy, while a minority received radiotherapy. As shown in Table 6, the treatment strategies included surgical intervention in 91.3% of CRC cases, chemotherapy in 90.1% of CRC cases, and radiotherapy in 15.1% of CRC cases. However, in some cases, the type of the surgery was unknown and may have corresponded to palliative resection of the primary tumor or of hepatic metastasis with curative intent or any other surgery. Likewise, the patient records did not reflect the type of chemotherapy treatment, and there was no distinction between neo-adjuvant and adjuvant therapy.

Regrettably, undertreatment was noted in four patients receiving FOLFOX and could have been prevented upon consultation with a clinical pharmacologist.

Chemotherapy among the 252 patients included several options, as shown in Table 7. FOLFOX is a combination of chemotherapy drugs used to treat CRC; it consists of folinic acid, fluorouracil, and oxaliplatin. FOLFIRI is the name of a chemotherapy combination that includes folinic acid, fluorouracil, and irinotecan. Some patients received other chemotherapy protocols, including the use of other small-molecule drugs by using monoclonal antibodies (mAbs) such as cetuximab, bevacizumab, and capecitabine. FOLFOX was more prevalent among patients, and physicians tend to prefer it over FOLFIRI.

Outcomes of treatment. The disease outcomes after treatment were categorized into six categories, namely, death, cure, disease progression, disease recurrence, undertreatment, or unknown outcomes. The results are presented in Table 8. The average follow-up time between the first diagnosis and the last visit was 3.25 ± 2.64 years. The minimum follow-up time was 30 days, and the maximum follow-up time was 13 years. The mortality of the disease is high, as most of the patients (41.3%) have unfortunately died.

Our regression analysis of disease outcomes (death, cure, progression, recurrence, undertreatment) against the type of therapy that the patient received was significant (*p* = 0.001). Patients who have received surgical treatment tend to have superior curative outcomes than patients who have received radiotherapy or chemotherapy.

Notably, neither FOLFOX nor FOLFIRI treatments had any significant effect on the disease outcomes, as suggested by the, having *p*-values of 0.7 and 0.13, respectively. These findings were compounded by the absence of information on whether FOLFOX and FOLFIRI treatments had been given as adjuvant therapy to increase cure rates or as a form of palliative treatment. As such, little can be said about the efficacy FOLFOX therapy (58 deaths, 44 disease progressions, 3 recurrences, 8 cures, and 4 unreported outcomes) and FOLFIRI treatment (60 deaths, 41 disease progressions, 2 recurrences, and 2 cures). Also, the use of mAb did not significantly alter patient outcomes. Finally, the number of cycles of any chemotherapy regimen in the current sample ranged between 1 cycle and 28 cycles. However, our analysis of the number of cycles with the survival of patients or better or worse outcomes was not statistically significant. Regrettably, undertreatment was noted in four patients receiving FOLFOX and could have been prevented upon consultation with a clinical pharmacologist. As such, little or nothing can be said about chemotherapy efficiency among the CRC patients.

Years of survival. The years of survival were collected in two ways: it was either clearly mentioned in the patient’s medical records or could be determined from the date of diagnosis and date of the last visit. However, in cases where the years of survival were unclear in the medical records, the current study calculated survival based on the difference between the date of diagnosis and the date of the last visit. The mean of survival was 1062 (±974) days.

Our regression analysis of days between last visit and diagnosis date and against the type of treatment received (i.e., surgery, chemotherapy, radiotherapy) was significant, with a *p*-value of 0.033 (Table 9).

Surgical treatment had a positive impact on increasing the days of survival, and it was significant (*p* = 0.021). Radiotherapy had a positive impact, also increasing the days of survival, but its impact was less positive than that of surgical treatment; however, the difference was not significant (*p* = 0.407). In addition, chemotherapy had a positive impact, but its was the lowest amongst all therapeutic options, and it was not significant (*p* = 0.096). The table below (Table 9) shows a regression analysis for the type of treatment and days of survival.

An χ-square analysis of the stage at diagnosis and the prognosis of CRC patients using existing data revealed that there is a significant difference (*p* < 0.05) between the stage at diagnosis and the disease outcome (Table 10). In the table below, the cure rates are surprisingly low, and only 23% of patients with stage III disease are shown as cured. In contrast, survival rates determined by the AJCC system have been reported to be >90% in stage I, 70–85% in stage II, 25–80% in stage III, and <10% in stage IV [21]. These survival rates do not coincide with our findings and potentially throw the staging of the patients into question.

## 4. Discussion

The current study aimed to capture the scope of colorectal cancer in the West Bank by studying the distribution of the cases in terms of colorectal cancer stages among Palestinian patients. Moreover, the study evaluated the prevailing CRC treatment methods and management strategies employed by physicians. Additionally, we also investigated disease progression and its relation to the treatment strategy, protocol of chemotherapy, and number of cycles. In addition, post-treatment disease outcomes among the sample were explored to relate them to the disease outcomes resulting from decisions made by physicians. A random sample of 252 patients from An-Najah University Hospital, Nablus, the West Bank, was included in the study. A retrospective review of medical records was performed to collect the required data to achieve the objectives of the study.

Colorectal cancer (CRC) in the West Bank. The current study gave an insight into the epidemiology of colorectal cancer in the West Bank. It showed that male patients with a mean age of 60.64 ± 11.4 years are more prone to develop CRC. Although these results are from one hospital in one city in the West Bank, the results coincide with the global epidemiology of CRC and especially that of CRC in the Arab World, as shown in the review by Arafa and Farhat [22]. As most CRC patients are of an older age, they tend to have more comorbidities. The current sample had many comorbid conditions, such as hypertension and diabetes mellitus; this can be related to the high prevalence of chronic disease in the West Bank [23].

Colorectal cancer stages among West Bank patients. The survival rate for patients with colorectal cancer is strictly correlated with the stage of the disease at diagnosis; the earlier the stage at diagnosis, the higher the chance of survival. Most patients in the current study’s sample were of stage IV (63%), and thus, this should be considered as a sign that we need to raise the awareness of CRC disease screening, as patients stay undiagnosed for a very long period until they reach the worst stage, which will highly increase the mortality rate of the disease. Many studies have also revealed that most patients present themselves late [24,25,26]. However, it should be noticed that NUH is a tertiary hospital where some advanced cancer cases from Gaza Strip or other hospitals in the West Bank are referred to for medical treatment. This could explain, in part, the very high percentage of advanced stage IV patients.

Palestine’s Ministry of Health publishes annual health reports stating the distribution of cancer diseases among Palestinians, and they provide large data sets; however, they do not provide data on the stages of cancer, treatments, and survival outcomes of patients.

The current study found that 41.3% of the patients died even after treatment, and 38.1% developed disease progression, with a low cure rate of only 11.9%. The high mortality rate due to colorectal cancer in the West Bank could be correlated with the findings on the stages of colorectal cancer at presentation, which emphasizes the need for the development of a colorectal cancer screening program at a national level. Colorectal cancer screening programs can detect and diagnose cases much earlier and provide health benefits; with their implementation, we could anticipate a reduction in the incidence of colorectal cancers diagnosed at late stages in the West Bank, thus improving survival.

This underscores the urgency of implementing an early detection program for colorectal cancer in the West Bank (for example, in high-risk populations such as long-term users of proton pump inhibitors [27]). The observation that colon cancer is frequently diagnosed at an advanced stage highlights the pressing need for accessible screening methods such as fecal occult blood testing or Cologuard. These non-invasive screening options could play a vital role in detecting colorectal cancer at its early stages, when treatment is most effective. By expanding screening programs and improving access to colonoscopy services where appropriate, healthcare providers could significantly impact patient outcomes by diagnosing colorectal cancer earlier, ultimately reducing mortality rates.

Management of colorectal cancer. The management of colorectal cancer depends on whether it is rectal cancer or colon cancer in the first place. In the current study’s sample, 27.3% had rectal cancer, and its treatment differs in early stages, as anatomic conditions are distinctive from the rest of the colon, and local recurrence is a major problem for morbidity and quality of life. Surgical therapy was found to be the most common mode of treatment among all patients. Also, chemotherapy had almost the same percentage, which means that most patients who receive chemotherapy either end up undergoing surgery or start with surgery and end with chemotherapy. There was no clear trend in treatment strategy among patients with CRC in the West Bank. We found that patients receive FOLFOX, FOLFIRI, or any combination of their constituents in addition to a monoclonal antibody (e.g., cetuximab or bevacizumab), or, more simply, other protocols using either capecitabine alone, irinotecan, bevacizumab alone, or their combinations. The treatment outcomes were not as bright as the physicians expected, presumably because the mortality rate is high. This does not mean that physicians are not truly following the international guidelines. However, there should be distinctive centers for oncology and the treatment of cancer patients where the patients receive treatment from highly specialized physicians and consultants, as well as healthcare staff that are well trained on how to follow these guidelines, especially clinical pharmacists.

There are numerous studies that have confirmed the role of clinical pharmacists in hematology/oncology and their contribution to better therapy outcomes and improving morbidity and mortality rates [28,29]. In the West Bank, the role of clinical pharmacists is almost negligible [30]. To increase survival likelihoods, it is highly recommended to include them in colorectal cancer healthcare teams in the West Bank.

This highlights the importance of optimizing treatment strategies for colorectal cancer patients in the West Bank, particularly regarding the lack of adherence to international guidelines and the need for specialized centers with comprehensive healthcare teams. Integrating clinical pharmacists into these teams could significantly enhance therapy outcomes and ultimately improve patient survival rates.

Disease outcomes. After treatment, the disease outcomes were categorized into six categories, namely, death, cure, disease progression, disease recurrence, undertreatment, or unknown outcomes. Despite the fact that the retrospective chart reviews were only for one year, unfortunately, the mortality rate was high (41.3%). It is known that the second most common cause of death in the West Bank is cancer, after cardiovascular diseases. Thus, 15.5% of the West Bank population dies because of cancer [16].

In the current study, a regression analysis of disease outcome (death, cure, progression, recurrence, undertreatment) against the type of therapy that the patient received was significant. Patients treated surgically tend to have better outcomes than patients that have received radiotherapy or chemotherapy. In addition, by comparing FOLFOX and FOLFIRI chemotherapy in terms of treatment outcomes, FOLFIRI protocols had higher death rates compared to FOLFOX protocols, which had higher cure rates; however, the difference was insignificant.

Colorectal cancer survival in the West Bank. Information on cancer survival is an important indicator of the cancer system’s effectiveness in detecting and treating cancer. Colorectal cancer (CRC) survival is highly dependent on the stage of disease at diagnosis. Theoretically, the diagnosis of the disease is the most crucial point that determines whether the patient is going to survive or not, i.e., the later the diagnosis, the worst the outcomes. This issue was investigated by (Pita-Fernández et al. [25], who concluded that a delay in the diagnosis of rectal cancer is linked to poor survival, while a delay in the diagnosis of colon cancer was not associated with poor survival; hence, these researchers believe that delays in diagnoses are not the sole factor leading to poor survival (Pita-Fernández et al., 2016 [25]). In the current study, our regression analysis of days between last visit and diagnosis date and the type of treatment received (chemotherapy, surgical treatment, radiotherapy) (R^2^ = 0.035) was significant (*p* = 0.033). A χ-square correlation analysis of the stage at diagnosis and the prognosis of CRC patients using existing data revealed that there is a significant difference (*p* < 0.05) between the stage at diagnosis and the disease outcome.

Surgical treatment had a positive impact on increasing the days of survival, and it was significant (*p* = 0.021). Radiotherapy has a positive impact on increasing the days of survival as well, but its impact was lower and less positive than that of surgical treatment; however, the difference was insignificant (*p* = 0.407). In addition, chemotherapy had a positive impact, but its impact was the lowest amongst all therapeutic options, and the difference was insignificant (*p* = 0.096). This could be explained by the fact that many patients with advanced metastatic stage IV disease are not eligible for surgery. Having surgery means that the tumor is operable, which may reflect a less advanced stage and better survival.

The gaps in the survival rates probably reflect the difference in the management practices among countries. A lack of cohesive practice guidelines for colorectal management and inadequate development to deal with the increasing demand for diagnostic, therapeutic, and follow-up care interventions could be reasons for the lower survival rate in the West Bank.

Genetic counseling. Genetic counseling is crucial, especially considering that among the 252 colorectal cancer (CRC) patients discussed earlier, 40 had both colon and rectal cancer. This concurrence raises the possibility of inherited susceptibility genes for cancer. While genetic counseling is available in the West Bank, there is a need to expand molecular diagnostics in this region by including, for example, reflex testing for mismatch repair proteins. This expansion would involve implementing advanced genetic testing techniques to better understand the genetic basis of colorectal cancer cases, thus facilitating proactive management and prevention strategies.

Strengths and limitations. To the best of our knowledge, this is the first report from the West Bank that evaluates the stages and outcomes of colorectal cancer, along with treatment protocols. This study is a retrospective study, and as a limitation of all retrospective studies, the data can be considered insufficient since, upon documentation, it were incomplete. Clinical data regarding weight, height, and lab tests were not available. In addition, since the data were collected from one hospital in Nablus only, the study’s generalizability is weak, and further national studies need to be undertaken in order to generalize the results.

Conclusion. A high percentage of patients were diagnosed in advanced stages. The modes of treatment were generally adopted from international guidelines; however, the cure rates and cure outcomes were not high, and the disease mortality rate was high.

Recommendations. This study makes the following recommendations for the evaluation and treatment of colorectal cancer in the West Bank: (1) More efforts should be directed at increasing colorectal cancer awareness among the general public and at implementing preventative screening actions performed by primary care physicians. For example, occult blood tests and colonoscopies should be incorporated into routine screening practices. (2) Additional studies should investigate the actual application of chemotherapy protocols and substantiate patient history documentation. (3) Choices regarding chemotherapy protocols, drug selection, dosing, and frequency should involve clinical pharmacists, as advocated by the numerous records of patients under treatment.

## Figures and Tables

**Table 1:** Demographic information of patients.

		N	(%)	Total
Gender	Male	143	(56.7)	252
	Female	109	(43.3)	
Age (years)	average (STD *)	60.64 (±11.4)		
	min	27		
	max	86		
Marital Status	single	67	(26.6)	
	married	173	(68.7)	
	widowed	10	(4.0)	
	divorced	2	(0.8)	
Smoking	no	187	(74.2)	
	ex-smoker	28	(11.1)	
	yes	37	(14.7)	
Alcohol Consumption	no	251	(99.6)	
	yes	1	(0.4)	
Height (cm)	average (STD *)	167.4 (±12.4)		83
	min	150		
	max	185		
Weight (kg)	average (STD *)	74.3 (±3.1)		95
	min	33		
	max	120		

* standard deviation.

**Table 2:** Medical histories of patients.

Comorbid Disease	N
None reported	128
Hypertension	73
Diabetes Mellitus	64
Hypothyroidism	15
Ischemic Heart Disease	13
Asthma	5
Chronic Kidney Disease	4
Heart Failure	3
End Stage Renal Disease	3

**Table 3:** Stages of colorectal cancer at diagnosis.

	N	(%)
Stage I	3	(1.2)
Stage II	33	(13.1)
Stage III	57	(22.6)
Stage IV	159	(63.1)
Total	252	(100)

**Table 4:** TNM stages of colorectal cancer at diagnosis.

	N
T1	1
T2	40
T3	133
T4	43
M0	87
M1	161
N0	61
N1	125
N2	36
N3	2

**Table 5:** Association between gender and colorectal cancer and stages.

	Colon Cancer (N)		Rectal Cancer (N)		Stage (N)			
	No	Yes	No	Yes	I	II	III	IV
Female	11	98	79	30	1	13	21	74
Male	18	125	104	39	2	20	36	84
*p* value *	0.53		0.96		0.55			

* χ-square test.

**Table 6:** Treatment strategies of colorectal cancer (CRC) patients.

	N
Surgical	230
Radiotherapy	38
Chemotherapy	227

**Table 7:** Chemotherapy drugs of colorectal cancer (CRC) patients.

	N	(%)
FOLFOX ^a^	35	(13.8)
FOLFOX ^a^ + mAb ^c^	4	(1.5)
FOLFOX ^a^ + Zolendronic acid	1	(0.4)
FOLFIRI ^b^	17	(6.7)
FOLFIRI ^b^ + mAb ^b^	8	(3.1)
Capecitabine	28	(11.1)
Capecitabine + Oxaliplatin	68	(26.9)
Bevacizumab	18	(7.1)
Cetuximab	6	(2.3)
Regorafenib	1	(0.4)
Cisplatin + Etoposide	1	(0.4)
Gemcitabine + Oxaliplatin	1	(0.4)
Other combination	43	(17.1)
Unknown	21	(8.3)
Total	252	(100)

^a^ FOLFOX: folinic acid (“FOL”), fluorouracil (“F”), and oxaliplatin (“OX”). ^b^ FOLFIRI: folinic acid (“FOL”), fluorouracil (“F”), and irinotecan (“IRI”). ^c^ mAb: monoclonal antibody drug.

**Table 8:** Outcomes of all treatments.

	N	(%)
Death	104	(41.3)
Cure	30	(11.9)
Disease progression	96	(38.1)
Disease recurrence	7	(2.8)
Undertreatment	6	(2.4)
Unknown	9	(3.6)
Total	252	(100)

**Table 9:** Regression analysis of colorectal treatment and years of survival.

ANOVA	Sum of Squares	df	Mean Square	F	*p*-Value
Regression	8,212,897	3	2,737,632.33	2.954	0.033 ^b^
Coefficients	Unstandardized coefficients		Standardized coefficients	t	*p*-value
	B	Std. Error	Beta		
Surgery	500.6 ^a^	215.6	0.147	2.323	0.021
Radiotherapy	142.0 ^a^	170.8	0.053	0.831	0.407
Chemotherapy	347.9 ^a^	208.4	0.106	1.670	0.096

^a^ dependent variable: days between last visit and diagnosis date. ^b^ predictors: (constant), surgery, chemotherapy, radiotherapy.

**Table 10:** Outcomes after treatment according to stage at diagnosis.

	Stage I		Stage II		Stage III		Stage IV		
	N	(%)	N	(%)	N	(%)	N	(%)	N
Death	0	0	5	−4.8	6	−5.8	93	−89.4	104
Cure	3	−10	19	−63.3	7	−23.3	1	−3.3	30
Disease Progression	0	0	4	−4.2	35	−36.8	56	−58.9	95
Disease Recurrence	0	0	2	−28.6	4	−57.1	1	−14.3	7
Undertreatment	0	0	3	−50	3	−50	0	0	6
Unknown	0	0	0	0	2	−22.2	8	−88	10
Total	3	−1.2	33	−13.1	57	−22.6	159	−63.1	252

## Data Availability

This paper is a condensed and peer reviewed report of the MPharm thesis of the first authors, Ibrahim O Sawaid. The full thesis may be found at https://repository.najah.edu/items/2298b54e-e704-468e-90a8-2edb85da8e34 (accessed on 3 January 2024).

## Supplementary Materials

The following supporting information can be downloaded at: https://www.mdpi.com/article/10.3390/jcm13082284/s1, Table S1 shows the data collection form.

## Abbreviations

ANOVA—analysis of variance; BMT—bone marrow transplantation; CI—confidence interval; CKD—chronic kidney disease; CRC—colorectal cancer; CRP—C-reactive protein; CT—computed tomography, DM—diabetes mellitus; ESRD—end-stage renal disease; FOLFIRI—5-fluorouracil, leucovorin, irinotecan; FOLFOX—5-fluorouracil, leucovorin, oxaliplatin; HF—Heart Failure; HTN—hypertension; IHD—ischemic heart disease; IRB—institution review board; mAb—monoclonal antibody; MCV—Mean Corpuscular Volume; MRI—magnetic resonance imaging; NNU—Najah National University; NUH—Najah University Hospital; PA—Palestinian Authority; PL—Platelet; SPSS—Statistical Package for Social Sciences; TNM stage—Classification of Malignant Tumors; WBCs—white blood cells; WHO—World Health Organization.

## References

[1] Beaver K., Wilson C., Procter D., Sheridan J., Towers G., Heath J., Susnerwala S., Luker K. (2011). Colorectal cancer follow-up: Patient satisfaction and amenability to telephone after care. Eur. J. Oncol. Nursing..

[2] Zhivotovskiy A.S., Kutikhin A.G., Azanov A.Z., Yuzhalin A.E., Magarill Y.A., Brusina E.B. (2012). Colorectal cancer risk factors among the population of south-east siberia: A case-control study. Asian Pac. J. Cancer Prev..

[3] Parkin D.M., Bray F.I., Devesa S.S. (2001). Cancer burden in the year 2000. The global picture. Eur. J. Cancer..

[4] Winawer S.J. (2007). Colorectal cancer screening. Best Pract. Res. Clin. Gastroenterol..

[5] Ferlay J., Shin H.R., Bray F., Forman D., Mathers C., Parkin D.M. (2010). Estimates of worldwide burden of cancer in 2008: GLOBOCAN 2008. Int. J. Cancer.

[6] Ferlay J., Parkin D., Steliarova-Foucher E. (2010). Estimates of cancer incidence and mortality in Europe in 2008. Eur. J. Cancer.

[7] Rasool S., Kadla S.A., Rasool V., Ganai B.A. (2013). A comparative overview of general risk factors associated with the incidence of colorectal cancer. Tumor Biol..

[8] Marley A.R., Nan H. (2016). Epidemiology of colorectal cancer. Int. J. Mol. Epidemiol. Genet..

[9] Johnson C.M., Wei C., Ensor J.E., Smolenski D.J., Amos C.I., Levin B., Berry D.A. (2013). Meta-analyses of colorectal cancer risk factors. Cancer Causes Control.

[10] Jass J.R. (2002). Pathogenesis of colorectal cancer. Surg. Clin. N. Am..

[11] Jörgensen O.D., Kronborg O., Fenger C. (2002). A randomised study of screening for colorectal cancer using faecal occult blood testing: Results after 13 years and seven biennial screening rounds. Gut.

[12] Mandel J.S., Church T.R., Bond J.H., Ederer F., Geisser M.S., Mongin S.J., Snover D.C., Schuman L.M. (2000). The effect of fecal occult-blood screening on the incidence of colorectal cancer. N. Engl. J. Med..

[13] Singh S., Singh P.P., Murad M.H., Singh H., Samadder N.J. (2014). Prevalence, risk factors, and outcomes of interval colorectal cancers: A systematic review and meta-analysis. Am. J. Gastroenterol..

[14] Halahleh K., Gale R.P. (2018). Cancer care in the Palestinian territories. Lancet Oncol..

[15] Qumseya B.J., Tayem Y.I., Dasa O.Y., Nahhal K.W., Abu-Limon I.M., Hmidat A.M., Al–Shareif A.F., Hamadneh M.K., Riegert–Johnson D.L., Wallace M.B. (2014). Barriers to colorectal cancer screening in Palestine: A national study in a medically underserved population. Clin. Gastroenterol. Hepatol..

[16] Palestinian Authority Ministry of Health Health Annual Report, Palestine 2019. http://site.moh.ps/Content/Books/HYM2UGrm8hFDOPe1AW6z2W6ZDvbJbuYGykdfV6B1lEulthrx5QMAyC_5WFKDTWWGKW3O7rk4vgIUzRlhJdSYyQXxFKscP6Uqz3UhrxoWLcHlT.pdf.

[17] Bailony M.R., Hararah M.K., Salhab A.R., Ghannam I., Abdeen Z., Ghannam J. (2011). Cancer registration and healthcare access in West Bank, Palestine: A GIS analysis of childhood cancer, 1998–2007. Int. J. Cancer.

[18] Kharroubi A.T., Abu Seir R.Y. (2016). Cancer Care in Palestine. Cancer Care in Countries and Societies in Transition.

[19] Abu-Rmeileh N.M.E., Gianicolo E.A.L., Bruni A., Mitwali S., Portaluri M., Bitar J., Hamad M., Giacaman R., Vigotti M.A. (2016). Cancer mortality in the West Bank, Occupied Palestinian Territory. BMC Public Health.

[20] Patel S.G., Karlitz J.J., Yen T., Lieu C.H., Boland C.R. (2022). The rising tide of early-onset colorectal cancer: A comprehensive review of epidemiology, clinical features, biology, risk factors, prevention, and early detection. Lancet Gastroenterol. Hepatol..

[21] Hong Y., Kim J., Choi Y.J., Kang J.G. (2020). Clinical study of colorectal cancer operation: Survival analysis. Korean J. Clin. Oncol..

[22] Arafa M.A., Farhat K. (2015). Colorectal Cancer in the Arab World—Screening Practices and Future Prospects. Asian Pac. J. Cancer Prev..

[23] Husseini A., Abu-Rmeileh N.M., Mikki N., Ramahi T.M., Ghosh H.A., Barghuthi N., Khalili M., Bjertness E., Holmboe-Ottesen G., Jervell J. (2009). Cardiovascular diseases, diabetes mellitus, and cancer in the occupied Palestinian territory. Lancet.

[24] Korsgaard M., Pedersen L., Laurberg S. (2008). Delay of diagnosis and treatment of colorectal cancer—A population-based Danish Study. Cancer Detect. Prev..

[25] Pita-Fernández S., González-Sáez L., López-Calviño B., Seoane-Pillado T., Rodríguez-Camacho E., Pazos-Sierra A., González-Santamaría P., Pértega-Díaz S. (2016). Effect of diagnostic delay on survival in patients with colorectal cancer: A retrospective cohort study. BMC Cancer..

[26] Delpeuch A., Leveque D., Gourieux B., Herbrecht R. (2015). Impact of clinical pharmacy services in a hematology/oncology inpatient setting. Anticancer Res..

[27] Duarte R.F., Labopin M., Bader P., Basak G.W., Bonini C., Chabannon C., Corbacioglu S., Dreger P., Dufour C., Gennery A.R. (2019). Indications for haematopoietic stem cell transplantation for haematological diseases, solid tumours and immune disorders: Current practice in Europe, 2019. Bone Marrow Transpl..

[28] Sawaid I.O., Samson A.O. (2024). Proton Pump Inhibitors and Cancer Risk: A Comprehensive Review of Epidemiological and Mechanistic Evidence. J. Clin. Med..

[29] Moukafih B., Abahssain H., Mrabti H., Errihani H., Rahali Y., Taoufik J., Chaibi A. (2021). Impact of clinical pharmacy services in a hematology/oncology ward in Morocco. J. Oncol. Pharm. Pract..

[30] Naseef H., Amria A., Dreidi M. (2020). The acceptance and awareness of healthcare providers towards Doctor of Pharmacy (Pharm D) in the Palestinian health care system. Saudi Pharm. J..

