# Supplementary material for: Evaluation of Stages, Treatment Protocols, and Outcomes of Colorectal Cancer among West Bank Patients
Source: J Clin Med. 2024 Apr 15;13(8):2284. doi: 10.3390/jcm13082284 (PMC11051243; doi:10.3390/jcm13082284)
Supplement: Supplementary file 1 [file jcm-13-02284-s001.zip › jcm-2811705-supplementary.pdf]

Journal of Clinical Medicine

MDPI

**Supplementary Table S1. Data Collection Form**

| | | | |
|---|---|---|---|
| **MEDICAL RECORD** #_______________ | **GENDER** Male Female | **DATE OF BIRTH** _________ **Age**_________ | |
| **BLOOD GRP** ___________ | **HEIGHT** ____________ | **WEIGHT** ____________ | **BMI** _____________ |

| | | | | |
|---|---|---|---|---|
| **MARITAL STATUS** | Single | Married | Divorced | Widowed |
| **EDUCATION** | No formal Edu. | Primary | Secondary | Tertiary |
| **SMOKING** | Smoker | Non-Smoker | Ex-smoker | |
| **ALCOHOL** | Drinker | Ex-drinker | Non-drinker | |
| **NUTRITION** | Well Nourished | Malnourished | Vegetarian | |
| **Allergy** | None | Allergic to _________________ | | |

| | | | | | |
|---|---|---|---|---|---|
| **DISEASE HISTORY** | Diabetes | Dyslipidemia | Hypertension | IHD | ____________ |
| | Stroke | Heart Failure | Arrhythmia | IBD | ____________ |
| | Asthma | IBS | L. Cirrhosis | R. Arthritis | ____________ |
| | COPD | CKD | Gout | Osteoporosis | ____________ |
| **FAMILY HISTORY** | Cancer (Type ___________) | NONE | | | |

**B: COLORECTAL CANCER CHARACTERISTICS**

| | |
|---|---|
| **Colon cancer** | Yes No |
| **Rectum cancer** | Yes No |
| **Stage (TNM)** | |
| **Diagnosis date** | |
| **Last visit date** | |
| **Treatment method** | Surgical Radiotherapy Chemotherapy |
| **Medical treatment protocol** | FOLFOX FOLFIRI Other_______________________ |
| **Number of cycles completed** | |

**C: LAB RESULTS (last readings)**

| Renal function test | | | | | CBC | | | | | Other | | | | |
|---|---|---|---|---|---|---|---|---|---|---|---|---|---|---|
| **Na** | | | | 135-145 mmol/L | **RBC** | | | | 4-11 x103/mm3 | **R.B Glu** | | | | |
| **K** | | | | 3 -5 mmol/L | **WBC** | | | | 4-6.2 x103/mm3 | **HbA1c** | | | | |
| **Urea** | | | | 1.7 – 8.3 mmol/L | **HGB** | | | | 11-18 g/dL | **INR** | | | | |
| **Cr** | | | | 53-97 µmol/L 44-80µmol/L | **HCT** | | | | 35-55% | **CRP** | | | | |
| **Cr Cl** | | | | | **MCV** | | | | 80-100 | | | | | |
| **Uric acid** | | | | 210-420 µmol/L | **PLT** | | | | 150-400 x103/mm3 | | | | | |
| **Mg** | | | | 0.7-1.05 | | | | | | | | | | |
| **Ca** | | | | 2.3-2.5mmol/L | | | | | | | | | | |
| **Phosp** | | | | 0.9-1.3 mmol/L | | | | | | | | | | |

**D: Home MEDICATIONS**

| Drug | Frequency | |
|---|---|---|
| | | |
| | | |
| | | |
| | | |
| | | |
| | | |
| | | |

**Outcomes of treatment:** **cure** **death** **recurrence** **not known**

**Years of survival after diagnosis:________________**
